# Metabolic Stress Adaptations Underlie Mammary Gland Morphogenesis and Breast Cancer Progression

**DOI:** 10.3390/cells10102641

**Published:** 2021-10-02

**Authors:** Chun-Chao Wang

**Affiliations:** 1 Institute of Molecular Medicine, National Tsing Hua University, Hsinchu 30013, Taiwan; ccwang@life.nthu.edu.tw; Tel.: +886-3-516-2589; 2 Department of Medical Science, National Tsing Hua University, Hsinchu 30013, Taiwan

**Keywords:** mammary gland morphogenesis, terminal end bud, 3D spheroid culture, breast cancer progression, metabolic stress

## Abstract

Breast cancers display dynamic reprogrammed metabolic activities as cancers develop from premalignant lesions to primary tumors, and then metastasize. Numerous advances focus on how tumors develop pro-proliferative metabolic signaling that differs them from adjacent, non-transformed epithelial tissues. This leads to targetable oncogene-driven liabilities among breast cancer subtypes. Other advances demonstrate how microenvironments trigger stress-response at single-cell resolution. Microenvironmental heterogeneities give rise to cell regulatory states in cancer cell spheroids in three-dimensional cultures and at stratified terminal end buds during mammary gland morphogenesis, where stress and survival signaling juxtapose. The cell-state specificity in stress signaling networks recapture metabolic evolution during cancer progression. Understanding lineage-specific metabolic phenotypes in experimental models is useful for gaining a deeper understanding of subtype-selective breast cancer metabolism.

## 1. Metabolic Regulation in Mammary Gland Morphogenesis

### 1.1. Mammary Gland Morphogenesis

**Figure 1:**
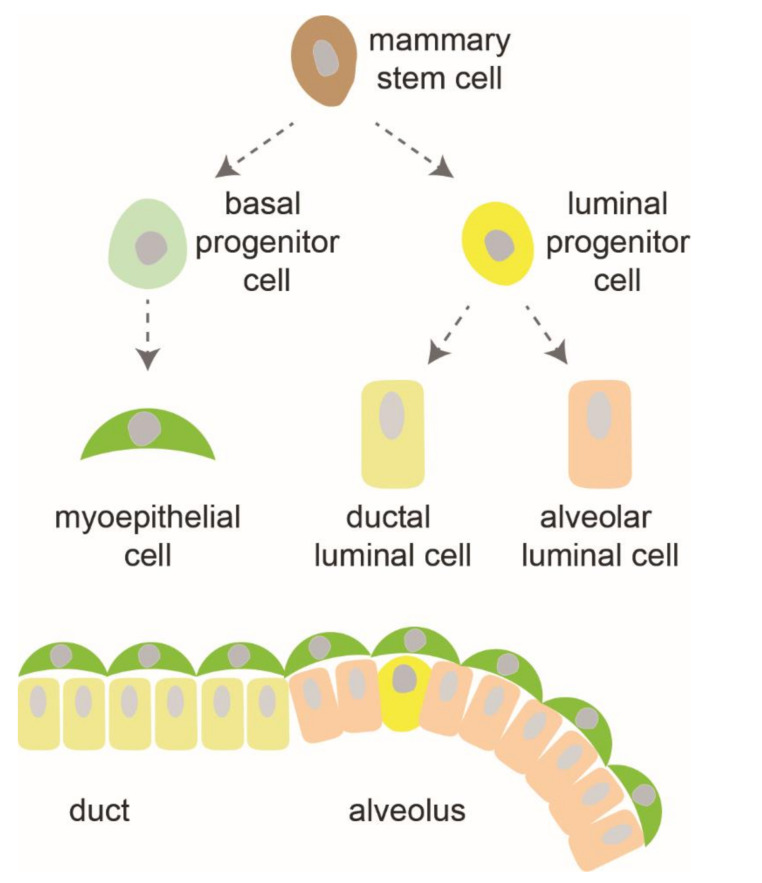
Cell differentiation hierarchy in mammary gland development.

The mammary gland is a unique organ that shows a remarkable regenerative ability [1,2]. Its development and homeostasis involve extensive expansion and reorganization throughout life. In the embryo, fetal mammary stem cells, the earliest cells shown in mammary morphogenesis, are bipotent cells with both basal and luminal gene expression profiles (Figure 1). When the breast-mammary epithelium is specified in the early embryo, it forms a mammary bud that consists of a sphere of arrayed mammary epithelial cells. These cells, emanating from the nipple region, invaginate into the stromal fat pad, and begin the initial round of branching growth to form the rudimentary ductal tree. The initial ductal structures in female mice remain largely quiescent until puberty. During puberty, the mammary gland undergoes a second round of expansion, proliferation and ductal branching morphogenesis, to form a developed mammary ductal tree. The mature mammary gland is composed of basal, luminal progenitor, and more differentiated luminal cells [3]. Ductal branching morphogenesis continues as a result of elevated concentration of hormones during pregnancy, forming a series of branching ducts that terminate in clusters of milk-secreting alveoli (lobules) in a process called alveologenesis. Alveolar epithelial cells differentiate, polarize, and form the milk-secreting alveoli. After lactation, alveolar cells are rapidly cleared by apoptosis with tissue remodeling through involution process, and mammary glands return to their pre-pregnant state [4]. The ability to undergo repeated rounds of alveologenesis, lactation, and involution during successive pregnancies reflects the regenerative capabilities of mammary glands.

### 1.2. Terminal End Buds

During puberty, formation of the branched network of epithelial ducts is orchestrated by the bulbous invasive structures called the terminal end buds (TEBs) [5,6]. Most knowledge of TEBs comes from studies on rodents; however, samples from teenage women indicate TEB structures are not different from those of rodents [7]. TEBs arise at the tips of the ducts. They direct the elongation of ducts to produce the main mammary ductal tree. By the time the epithelial network has invaded the entire fat pad, the TEBs regress and are replaced by terminal duct structures, which are composed of terminal ductules (acini).

TEBs consist of two compartments [8]. The outer basal layer is a single-cell layer composed of rapidly dividing cap cells that contact with microenvironment directly. The inner multi-cellular layer is known as the body cell layer. As the duct elongates, cap cells differentiate into myoepithelial cells that envelop the ducts. The cap cells and myoepithelial cells deposit the extracellular matrix (ECM) proteins and assemble the adhesive basement membrane. The body cells adjacent to the basal layer become polarized and differentiate into luminal progenitors. The inner body cells undergo apoptosis and are removed by luminal phagocytes, a process known as lumen formation [9]. During alveolar morphogenesis, luminal cells differentiate into alveolar cells that are capable of producing milk.

When the mammary gland undergoes dynamic developmental changes, TEBs are continually proliferating and regressing in response to different microenvironments: each promotes branching, elongation, alveolar differentiation, or epithelial apoptosis at different developmental stages [10]. At the leading tip, the TEB cells uniquely come into direct contact with microenvironmental cues in the stroma that is made up of several types of cells and signaling factors. Three cell types (fibroblasts, eosinophils, and macrophages) are reported to be important for ductal elongation. Fibroblasts and eosinophils localize around the advancing tips of TEBs. Fibroblasts are responsible for producing growth factors and facilitating production and degradation of ECM components, and are important for ductal elongation [11]. Eosinophils are recruited by eotaxin-secreting TEB cells and are involved in branching. Macrophages found at the neck of the TEBs help ECM remodeling [12]. When infiltrating into the body cell layer of TEBs, macrophages contribute to lumen formation.

Signal factors required for mammary gland development include hormones and growth factors. The endocrine hormone estrogen from the ovaries stimulates ductal growth through indirect paracrine mechanisms [13]. Estrogen binds its main receptor, estrogen receptor alpha, in luminal stromal cells, which are then responsible for the production of growth factors around TEBs. The pituitary growth hormone acts on fibroblasts around TEBs to produce insulin growth factor, which promotes TEB formation. The pituitary hormone prolactin stimulates alveolar differentiation and promotes alveolar cells to secrete milk. Several growth factors and ECM ligands from stroma provide supportive signaling for ductal development by interacting with epithelial cells through their receptors, including members of the epidermal growth factor receptor family, fibroblast growth factor receptors family, and integrin family. Transforming growth factor beta (TGF-β), however, is mainly produced by epithelial cells, and then stored in ECM as a latent form [14,15]. Activated TGF-β inhibits the proliferation of TEB cells. In stromal cells, TGF-β signaling acts as a driver of ECM deposition, which contributes to TEB regression. 

### 1.3. Terminal End Bud as a Model for Breast Cancer

As mentioned above, instructive signals required for proper mammary morphology have been well studied. Some other mechanisms underlying mammary morphogenesis could cause cellular damage or disease; however, are still areas of ongoing investigation. Many biological processes involved in TEB growth are required for the mammary tumor development. TEBs show invasive ability. They are able to create their own blood supply through expressing vascular endothelial growth factor or recruiting angiogenesis-related stromal cells (macrophages and endothelial cells) [16]. The TEBs can therefore be used as a model for studying the functions related to breast cancer. 

The TEB represents a stem cell niche. TEB cells function as either highly proliferative stem and progenitor cell lineages or pro-apoptotic cells that are heterogeneous and drive dynamic morphogenesis at developmental stages [17,18]. Cap cells are thought to be a reservoir for stem cells that have the ability to undergo symmetric divisions and asymmetric self-renewal. Cap cells are precursors to the basal layer. Dislocated cap cells (or cap-in-body cells) are found in the body cell layers [19], and have been considered to contribute to luminal lineage too [17,20]. Recent clonal analysis and lineage tracing studies, however, suggest that cap cells are lineage-restricted and contribute only to the basal layer [21,22]. Dislocated cap cells undergo apoptosis that could be rescued by WNT pathway, a pathway that is activated in breast cancer stem cells. Targeting WNT signaling may inhibit self-renewal of cancer stem cells. 

TEB is a good model for studying the stratified epithelial organization. Simple ductal epithelium is polarized and quiescent. It stratifies to form TEB during puberty. The TBE cells are highly proliferative, and have reduced apicobasal polarity and few intercellular junctions [23]. Stratification initiates from the asymmetric vertical apical divisions, resulting in generation of unpolarized internal cells. The stratification and polarity loss in the body layer is similar to morphology of premalignant breast lesions. 

### 1.4. Lineage-Specific Stress Signaling in Mammary Epithelium Development

The adult mammary gland consists of two layers of cells. In outer basal layer, myoepithelial cells directly contact the basement membrane. The inner luminal cells are polarized along the apical-basal axis. Single-cell RNA sequencing of primary human breast epithelial cells reveals the existence of three epithelial cell types: basal, mature luminal cells, and luminal progenitor [3]. Breast tissue from *BRCA1* mutation carriers harbors an aberrant population of luminal progenitor cells [24]. *BRCA1* has a role in elimination of R-loops, RNA–DNA hybrid structures that form during transcription. Defects in regulatory factors that resolve R-loops (such as *BRCA1*) can lead to genomic instability. Notably, *BRCA1* mutation-associated R-loop accumulation is commonly observed in luminal progenitors [25]. Aberrant R-loop accumulation, however, is not found in basal cells from BRCA1 mutant specimen. Luminal lineage-specific R-loop accumulation may contribute to BRCA1-associated tumorigenesis.

In addition to the difference in molecular states across the mammary lineages, divergent stress responses and metabolic identities are observed [26,27]. Basal cells isolated from human mammary epithelial cells have low reactive oxygen species (ROS) levels. In contrast, the matching luminal progenitors contain more mitochondria, and have higher levels of ROS production and uptake of O_2_. ROS overload promotes damage to DNA and other macromolecules in cells. The luminal progenitor cells display how to make use of multiple glutathione-independent antioxidant mechanisms to survive the high ROS level. A recent proteomics study of three epithelial cell populations illustrates distinct metabolic protein abundance [26]. Activated pathways in luminal progenitor cells are related to oxidation phosphorylation, which may represent the metabolic features necessary for the function of luminal progenitors.

The more differentiated, mature alveolar luminal cells have the ability to produce and secrete milk. The main macromolecular constituents of milk are lipid (~30%) and protein (~12%) [28]. During the transition from pregnancy to lactation, small lipid droplets and milk proteins can be observed within luminal alveolar cells. The increased demand in production of milk proteins and lipogenic enzymes could accumulate unfolded, misfolded or aggregated proteins in the endoplasmic reticulum (ER) of alveolar cells. To cope with ER stress, cells activate the unfolded protein response (UPR) program, which is initiated by three independent pathways: the protein kinase RNA-like endoplasmic reticulum kinase (PERK), X-box binding protein 1 (XBP1), and ATF6 signaling. In alveolar cells, lipogenesis is reported to be regulated by the PERK arm of the UPR [29]. The XBP1 arm of UPR is required for synthesis of major protein components of milk. These findings implicate stress tolerance and response, the mechanisms that are usually active in breast cancer, as physiologically relevant regulators of the mammary gland development and lactation.

## 2. Metabolic Regulators Heterogeneously Activated in 3D MCF10A Spheroids

Breast epithelial cells normally undergo an apoptotic process, termed anoikis, upon loss of attachment to ECM or neighboring cells [30,31,32,33]. Breast tumor cells, however, acquire anoikis resistance to survive in the luminal space of mammary acini, where normal epithelial cells are cleared from the lumen [34]. Three-dimensional (3D) spheroid structures of nonmalignant human breast epithelial MCF10A cells, cultured with reconstituted basement membrane ECM, have been used to mimic mammary gland acini [35]. Spheroid was formed under the influence of ECM components, beginning with clearance of the inner matrix-deprived cells to generate a hollow lumen, followed by polarization of the outer matrix-attached cells. Previous studies demonstrate that ECM components regulate metabolic activity [34]. In normal cells, matrix deprivation causes metabolic defects (loss of glucose uptake, ATP deficiency, and low availability of reducing equivalents (reduced glutathione/nicotinamide adenine dinucleotide phosphate (NADPH)). Matrix detachment generates high reactive ROS-detoxifying metabolites (such as glutathione) that cause oxidative stress in cells and induce anoikis. Introduction of oncogenes into MCF10A cells results in lumen-filled spheroids that resemble filled alveoli characteristic of breast premalignancy. The mechanism of antioxidant restoration and ATP generation are relevant to cancer cell survival in the disrupted ECM microenvironments.

Normal epithelial cells lack the genetic heterogeneity. However, they do perform the mechanisms of developmental plasticity and stress response by which nongenetic intratumoral heterogeneity may arise during the cancer evolution. Single-cell profiling has been used to examine the gene expression programs activated during 3D breast epithelial acinar morphogenesis [36]. The global analysis identifies *JUND* transcription factor, as well as a transcript cluster containing genes that have strong connection with oxidative stress responses and ER stress (*SLC25A28* [37], *BCL2L13* [38], *FAF2* [39], *PRDX4* [40], *FAM120A* [41], *SERP1* [42], and *FOXO1*). SLC25A28, a mitochondrial iron importer, was reported to mediate metabolic reprograming in tumorigenesis [37]. BCL2L13, the mitochondrial mitophagy receptor, can increase oxidative phosphorylation and provides mitochondrial quality control [38]. In response to environmental stressors, stress granules arise in the cytoplasm and are disassembled after the stress is relieved. The ER-associated protein FAF2 (FAS-associated factor 2) recruits stress granules and promotes their disassembly [39]. PRDX4 is the only PRDX (peroxiredoxins) family member located within the ER. Overoxidation of PRDX4 acts as inducers of the UPR and ER oxidative stress [40]. SERP1 is a γ-secretase activator in the cells experiencing ER stress [42]. The stress-response program is induced heterogeneously in single ECM-attached cells in 3D culture, suggesting that individual cells might face stressful niches during morphogenesis. 

FOXO1 transcription factor is a critical regulator of mammary stem cell homeostasis [21]. In pubertal TEBs, cap cells show cytoplasmic localization of the FOXO1, where nuclear FOXO1 is observed in dislocated cap-in-body cells, suggesting that FOXO1 mediates cell-cycle arrest and apoptosis. In basal-like MCF10A acini grown in 3D culture, single-cell regulation of FOXO1 function protects cells from oxidative stress that is caused by RUNX1 silencing. FOXO1 may cooperate with the tumor suppressing RUNX1 signaling to maintain an acute state of oxidative stress that causes proliferation arrest in normal 3D morphology and in triple-negative breast cancer cells.

JUND (jun D proto-oncogene) transcription factor regulates genes involved in antioxidant defense, H_2_O_2_ metabolism, and tumor angiogenesis [43]. During MCF10A acinar development, two transcriptional regulatory states defined by JUND and TGFBR3 (transforming growth factor β receptor 3) are anticorrelated at single-cell level. This signaling–transcription circuit modulates tissue architecture and may function in basal-like premalignancies [32]. JUND expression program contains a PAM50 basal-like gene, KRT5 [44] along with stress response genes (*CDKN1A* [45], *HSPE1* [46], and *MUS81* [47]). CDKN1A, the cyclin-dependent kinase inhibitor p21, is induced by p53 in response to DNA damage [45]. CDKN1A causes G1 arrest, mediating p53-dependent tumor suppression. During ER stress, p53 pathway, however, suppresses CDKN1A, which promotes G2 arrest and sensitizes cells to genotoxic drug-induced DNA damage. The heat shock protein HSPE1 is localized to mitochondria, and participates in protein folding and misfolded protein degradation [46]. The release of HSPE1 in response to stress accelerates caspase activation. In mammalian cells experiencing replication stress, the nucleolytic activity of MUS81 endonuclease is required for the disassembly and restart of the replication machinery. MUS81 thus promotes replication stress tolerance and sustains survival of BRCA2-deficient cells [47]. The genes in JUND cluster were among the predictors of response to chemotherapy-induced stress of breast cancer [48].

The spatially heterogeneous trigger of JUND cluster genes in MCF10A spheroids is coordinated by two stress-responsive transcription factors NRF2 (nuclear factor erythroid 2 like 2, *NFE2L2*) and p53 (*TP53*) [49]. NRF2 and p53 play important roles in oxidative stress handling in normal mammary epithelia. Cancer cells rewire metabolic networks for a steady source of ATP, which causes the production of metabolic byproduct ROS. In tumor 3D spheroids, inner, matrix-deprived cells that are under high oxidative stress, show reduced survival potential upon *NFE2L2* silencing [50]. Deletion of the KEAP1, the negative regulator of NRF2, enhances tumor cell survival. NRF2 induces the expression of antioxidants and promotes production of glutathione and NADPH. These findings suggest a broader role of NRF2 in tumor metabolic reprogramming.

## 3. Metabolic Reprogramming Governs Cancer Progression

**Figure 2:**
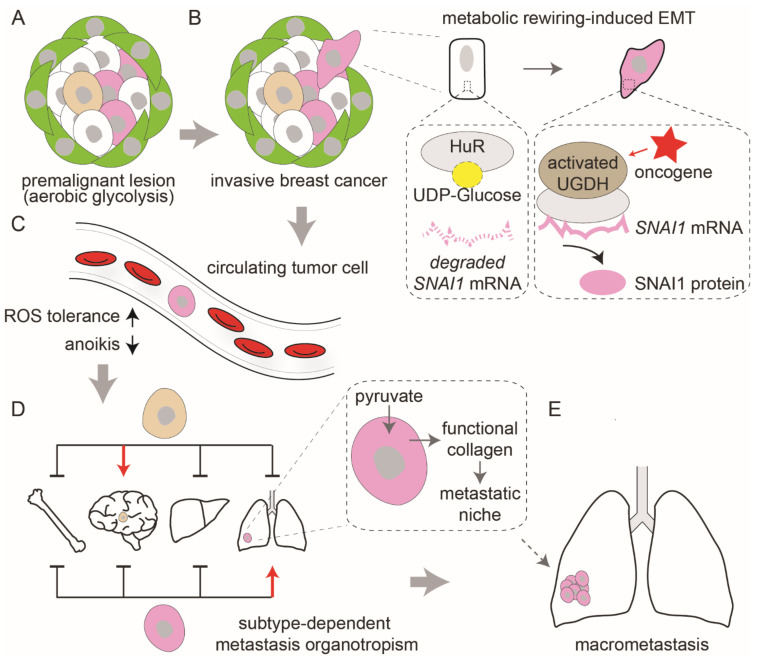
Tumor metabolic rewiring supports the metastatic cascade. (**A**) The Warburg effect is commonly observed in premalignant lesions. (**B**) Metabolites can affect cancer-cell epithelial–mesenchymal transition (EMT). In normal cells, binding of the HuR to a UDP-glucose destabilizes *SNAI1* mRNA. In tumor cells, oncogene activities enable UGDH to bind to HuR, stabilizing *SNAI1* mRNA and facilitating EMT process. (**C**) Circulating tumor cells develop resistance to oxidative stress and anoikis. (**D**) Breast cancer subtypes differ in their metastatic behavior. Subtype-selective pyruvate metabolism drives ECM remodeling in the lung metastatic niche. (**E**) The growth-promoting metabolic network is activated in macrometastases.

Metabolic plasticity determines tumor growth and metastasis. In contrast to high levels of aerobic glycolysis in primary tumor cells, mitochondrial oxidative phosphorylation promotes the metastatic seeding and tumor recurrence (Figure 2A) [51]. Different metabolic strategies may be required for tumor cells to go through stages of the metastatic cascade.

During the early stages of metastasis, overcoming the ECM detachment-induced metabolic changes and balancing redox homeostasis for survival are required for malignant cell survival. Detachment increases ROS by suppressing pentose phosphate pathway (PPP) signaling. Cancer cells may use their persistent PPP flux to mitigate mitochondrial ROS [52]. In the oxidizing microenvironment of bloodstream, oxidative stress and induction of defense gene expression (such as β-globin) are observed in circulating tumor cells (CTCs) (Figure 2C) [53]. Both ECM detachment and ROS exposure make resistance to oxidative stress a key factor in metastatic efficiency.

Metabolic reprogramming in tumor cells contributes to the migration and epithelial–mesenchymal transition (EMT). This morphological alteration involves reduced polarity and cell–cell junction, and cytoskeletal reorganization. EMT-associated transcription factors including *SNAI1*, *SNAI2*, *ZEB1*, and *ZEB2*, regulate the expression of epithelial and mesenchymal markers (*CDH1* and *VIM,* respectively). The uronic acid pathway metabolite, UDP-Glucose, suppresses the stability of mRNA encoding SNAI1, a EMT-associated transcription factor (Figure 2B) [54]. Oncogene-dependent activation of a metabolic enzyme UGDH (uridine 5′-diphosphate (UDP)–glucose 6-dehydrogenase) removes UDP-Glucose, resulting in the expression of *SNAI1* and EMT. Since EMT-associated proteins have high asparagine content, asparagine synthetase (ASNS) supports EMT through controlling asparagine bioavailability.

Colonization of distant organs is not a random process. The propensity of an organ to foster CTC is variable, and some tumor cells tend to colonize particular organs. The different nutrient accessibility in organs may determine the likelihood of cells to colonize. Some breast cancer cells metabolize the nutrient pyruvate, which is particularly available in the lungs, to drive ECM remodeling and niche formation (Figure 2D) [55]. These together support cancer cell metastasis in the lungs. In the lipid-poor environment of the brain, elevated fatty acid synthesis is observed in metastatic HER2 positive breast cancer cells [56]. This is an adaptation function of tumor cells to increase their local lipid availability. Similarly, in lipid-rich lymph node niche, metastatic tumor cells preferentially oxidize fatty acids as fuel, compared with the primary tumor cells [57].

After colonization, disseminated cancer cells enter a dormancy period. A proportion of these cells are reactivated and develop into macrometastasis (Figure 2E). In dormancy, cells persist in a slow-cycling or non-proliferative state, often as a result of dissemination of therapeutic intervention. Dormant tumor cells surviving oncogene ablation are highly dependent on mitochondrial oxidative phosphorylation or fatty acid oxidation to produce ATP for energy [58,59]. Both pathways increase the level of ROS. The subpopulation of breast cancer cells that survive after loss of HER2 is found to upregulate NRF2 in response to oxidative stress [60]. NRF2 signaling may be involved in the growth-promoting metabolic network in recurrent tumors.

## 4. Lineage-Specific and Subtype-Selective Stress Regulatory Networks in Mammary Gland and Breast Cancer

### 4.1. Lineage-Specific Metabolic Identities of Mammary Cells

Single-cell sequencing technologies have revealed epithelial lineage dynamics and lineage-specific regulatory circuits in 3D spheroids and in the developed mammary gland [36,61]. During embryonic development, bipotent fetal mammary stem cells have high levels of glycolytic enzymes and glycolytic end-product pyruvate, as in the tumor associated Warburg effect [61]. 

The mature mammary gland is composed of basal, luminal progenitor, and more differentiated luminal cells [3]. Basal cells tend to express abundant glycolytic enzymes. In contrast, luminal progenitors have higher levels of electron transport chain subunits and great capacity to handle ROS [26]. These three epithelial lineages have been proposed as the putative cell-of-origin of breast cancer subtypes. Basal cells can give rise to the mesenchymal claudin-low cancer cells, mature luminal cells to the hormone-receptor-expressing luminal A and B subtypes, and luminal progenitors to basal-like breast cancers [24]. The claudin-low and basal-like subtypes overlap with the TNBCs. Recent studies demonstrate that each breast cancer subtype retains metabolic signatures of its cell-of-origin, suggesting that distinct metabolic networks underlie the subtype-specific oxidative stress adaptations.

### 4.2. The Metabolic Reprogramming and Molecular Diversity of Primary Breast Cancers

Premalignant lesions progress to malignant tumors through cell proliferation and acquisition of mutations. By tumorigenic mutations, signaling involved in production of energy and macromolecules, and intracellular redox balance (such as *MYC* and *TP53*) is commonly reprogrammed. Breast cancer cells undergo a metabolic change, termed the Warburg effect, from oxidative phosphorylation to aerobic glycolysis. Thus, cancer cells consume glucose and produce lactate. In normal cells, p53 reduces the Warburg effect through expressing *TIGAR* (TP53 induced glycolysis regulatory phosphatase) [62] and *SCO2* (synthesis of cytochrome C oxidase 2) [63]. The p53 proteins control mitochondrial quality through expressing *PRKN* (parkin RBR E3 ubiquitin protein ligase) to eliminate unhealthy mitochondria [64]. By contrast, the Warburg effect is commonly observed in ductal carcinomas in situ (DCIS). In early intraductal breast cancers, the Warburg effect emerges in the poor metabolic conditions [65]. Cancer cells adapt to harsh microenvironments with more aggressive and dedifferentiated phenotypes.

Glutamine also serves as an important carbon source in cancer. Cleavage of glutamine by glutaminase generates glutamate. In turn, glutamate can be incorporated into the glutathione synthesis pathway or serves as a key substrate to produce reducing agent NADPH in the tricarboxylic acid (TCA) cycle. Both NADPH and glutathione act as key regulators of intracellular redox homeostasis. The p53 proteins induce the expression of *GLS2* (glutaminase 2), a regulator of glutamate and glutathione synthesis [66]. GLS2 controls ROS homeostasis and contributes to the tumor suppression function of p53. Mutant p53 increases glucose uptake through promoting the membrane translocation of GLUT1 (glucose transporter 1) [67]. This stimulates the Warburg effect in cancer cells.

Primary breast cancers can be divided into four molecular subtypes: luminal A (hormone-receptor-positive, HER2-negative), luminal B (hormone-receptor-positive, HER2-positive), HER2-enriched (hormone-receptor-negative, HER2-positive), and TNBC (hormone-receptor-negative, HER2-negative) breast cancers. The distinctive genomic alterations are associated with the molecular subtypes. TNBC, compared with other subtypes, expresses a higher level of *MYC* proto-oncogene [68]. *MYC* is associated with expression of glucose metabolism genes in TNBC, but not in other breast cancer subtypes [69]. In addition, *MYC* hyperactivation activates the XBP1 arm of the UPR for coping with proteotoxic stresses in TNBC cells [70]. The diverse metabolism in different molecular subtypes of breast cancer warrants further investigation.

### 4.3. TNBC-Specific Metabolic Signatures

In normal breast epithelial tissue and basal-like premalignancy, NRF2–p53 coordination provides an oxidative stress-handling network [49]. NRF2-mediated tolerance in premalignancy may permit the emergency of missense p53 mutations that drive the progression of TNBC. In cancer cells, oncogenic p53 mutant acts as homeostatic factors and facilitates tumor adaptation to oxidative, proteotoxic, and metabolic stresses. Nearly 80% of TNBC cases harbor mutations in the p53 gene [68]. When TNBC cell lines experience endogenous or chemotherapy-induced ER stress, mutant p53 activates the pro-survival ATF6 arm of the UPR [71]. Mutant p53 can also enhance N-glycoprotein folding in the ER through expressing its target gene *ENTPD5* (ectonucleoside triphosphate diphosphohydrolase 5) [72]. Mutant p53 contributes to cellular adaptation to proteotoxic ER stress. 

Different from other subtypes of breast cancer, TNBCs are characterized by elevated aerobic glycolysis and lower oxidative phosphorylation rate (the Warburg effect). The high abundance of GLUT1 (glucose transporter 1, encoded by *SLC2A1*), a factor for glucose uptake, is regulating TNBC cell metabolism [73]. Once transported into cells, glucose is converted to pyruvate and then to lactate. This glycolytic process is regulated by HDAC6 (histone deacetylase 6), which interacts with several key glycolytic enzymes and promotes glycolysis [74]. A key glycolytic enzyme, LDHA (lactate dehydrogenase A), catalyzes pyruvate to lactate. LDHA in TNBC cells controls the expression of granulocyte colony-stimulating factor and granulocyte macrophage colony-stimulating factor [75]. The TNBC glycolytic metabolism thus helps maintain the immunosuppressive tumor microenvironment.

Metastatic TNBC cells demonstrate energy dependency on mitochondrial fatty acid oxidation. This lipid metabolism regulation is induced by oncogene *MYC*, and may contribute to TNBC aggressiveness [76]. A prometastatic protein, CDCP1 (CUB-domain containing protein 1) inactivates ACSL (acyl CoA-synthetase ligase) enzyme, reducing lipid-droplet abundance and stimulating fatty acid oxidation [77]. Elevated fatty acid oxidation activates SRC oncoprotein through autophosphorylation at residue Y419 [78]. Inhibition of fatty acid oxidation abolishes TNBC metastatic properties. Aside from the crucial glucose metabolism in TNBCs, fatty acid oxidation produces sufficient ATP to fuel the metastatic cascade.

### 4.4. Metabolic Networks in Luminal Breast Cancer

Luminal breast tumors account for >70% of breast cancer incidence. The expression of estrogen receptor (ESR1, encoded by *ESR1* gene) defines luminal breast cancers. Binding of ESR1 to chromatin requires ESR1-cooperating factor GATA3, and ESR1 pioneer factor FOXA1 [79]; both are the key regulators of luminal differentiation. Under stress conditions, this luminal differentiation program is collectively regulated by kinase MSK1 (mitogen- and stress-activated kinase 1, encoded by *RPS6KA5* gene), thereby facilitating dormancy in breast cancer cells [80]. The results explain why differentiation and slow proliferation are common in dormant luminal tumor micrometastases. Top pathways in mature luminal metabolic network are related to glutamine and glutathione [26] and the metabolic distinction is likely regulated by GATA3. TNBCs and luminal breast cancers express *GLS* and *GLS2*, respectively, and both TNBCs and luminal-subtype cancer cells use glutamine to supply the TCA cycle [81]. The expression of *GLS2* in luminal breast cancer cells is through GATA3. *GLS2* is essential in luminal tumors, suggesting a critical role for glutamine metabolism in luminal breast cancer.

### 4.5. Metabolic Networks in HER2 Positive Breast Cancer

*ERBB2* gene amplification is observed in 20–30% of patients with breast cancer. Overexpression of ERBB2 in human breast cancers enhances glycolysis by increasing the expression of a glycolytic enzyme LDHA [82]. ERBB2 is a receptor tyrosine kinase, localized to the cell membrane. Translocation of ERBB2 into mitochondria has been reported in cancer cells that overexpress the receptor [83]. Mitochondrial ERBB2 enhances cellular glycolysis. In contrast, mitochondrial ERBB2 decreases oxygen consumption and electron transport chain activities, negatively regulates mitochondrial respiratory functions. ERBB2 thus drives HER2 positive breast cancer cells towards a Warburg effect-like phenotype.

Brain metastases occur only in 5–10% and 20% of which are luminal breast cancers and TNBCs [84]. However, the incidence of brain metastases is 30–55% in patients with HER2 positive breast cancers. Brain-predominant genes critical for the establishment of HER2 positive breast cancer brain metastases include a cytoskeletal protein βIII-tubulin (encoded by *TUBB3* gene) [85] and the fatty acid-binding protein 7 (encoded by *FABP7* gene) [86]. FABP7, a brain-specific intracellular lipid-binding protein, regulates fatty acid uptake and intracellular lipid droplet formation. FABP7 controls the expressions of metabolic and invasion-related proteins, supporting glycolysis and lipid droplet accumulation in HER2 positive breast cancer brain metastases. The unique HER2 positive metabolism is critical for the adaptation of cancers when seeded in the brain microenvironment.

## 5. Future Directions and Concluding Remarks

Recent works in breast cancer metabolism indicate that metabolic phenotypes evolve as cancer progresses. The metabolic heterogeneity is appreciated, expanding the scope of stress tolerance network dependency beyond the oncogenic metabolic pathways. Whether the heterogeneity is a manifestation of intrinsic differences in individual precursor cells or extrinsic stress factors imposed by the microenvironment remains an open question. Future research should further explore the location, multiplicity, and dynamics of cancer cells and their cell regulatory states that are associated with stress tolerance. This will require use of experimental models that allow transient and asynchronous stress responses to be observed in highly dynamic epithelial structures, and methods to assess metabolic phenotypes. Understanding the relevance of metabolic networks to mammary gland morphogenesis and breast cancer progression may gain a more therapeutically actionable perspective of cancer metabolism.

## Data Availability

Not applicable.
